# Comparative antioxidant activity and phytochemical content of five extracts of *Pleurotus ostreatus* (oyster mushroom)

**DOI:** 10.1038/s41598-024-54201-x

**Published:** 2024-02-15

**Authors:** Magdalene Eno Effiong, Chidinma Precious Umeokwochi, Israel Sunmola Afolabi, Shalom Nwodo Chinedu

**Affiliations:** 1 Department of Biochemistry, College of Science and Technology, Covenant University, Canaanland, PMB 1023, Ota, Ogun State Nigeria; 2 Covenant Applied Informatics and Communication Africa Centre of Excellence (CApIC-ACE), Covenant University, Canaanland, PMB 1023, Ota, Ogun State Nigeria; 3 Covenant University Public Health and Wellbeing Research Cluster (CUPHWERC) Covenant University, Canaanland, PMB 1023, Ota, Ogun State Nigeria

**Keywords:** *Pleurotus ostreatus*, Extraction solvents, Bioactive compounds, Antioxidants, Phytochemicals, Biochemistry, Microbiology

## Abstract

Reactive oxygen species reacts with numerous molecules in the body system causing oxidative damage, which requires antioxidants to ameliorate. *Pleurotus ostreatus*, a highly nutritious edible mushroom, has been reported to be rich in bioactive compounds. This study evaluated the comparative antioxidant activity and phytochemical contents of five extracts of *P. ostreatus*: aqueous (AE), chloroform (CE), ethanol (EE), methanol (ME) and n-hexane (HE). The phytochemical composition and antioxidant activity of the extracts were determined using standard in-vitro antioxidant assay methods. Results showed that the extracts contained alkaloids, tannins, saponins, flavonoids, terpenoids, phenolics, cardiac glycosides, carbohydrates, anthrocyanins, and betacyanins in varied amounts. CE had the highest flavonoid content (104.83 ± 29.46 mg/100 g); AE gave the highest phenol content of 24.14 ± 0.02 mg/100 g; tannin was highest in EE (25.12 ± 0.06 mg/100 g); HE had highest amounts of alkaloids (187.60 ± 0.28 mg/100 g) and saponins (0.16 ± 0.00 mg/100 g). Antioxidant analyses revealed that CE had the best hydroxyl radical activity of 250% at 100 µg/ml and ferric cyanide reducing power of 8495 µg/ml; ME gave the maximum DPPH activity (87.67%) and hydrogen peroxide scavenging activity (65.58%) at 500 µg/ml; EE had the highest nitric oxide radical inhibition of 65.81% at 500 µg/ml and ascorbate peroxidase activity of 1.60 (iU/l). AE had the best total antioxidant capacity (5.27 µg/ml GAE at 500 µg/ml) and ferrous iron chelating activity (99.23% at 100 µg/ml) while HE gave the highest guaiacol peroxidase activity of 0.20(iU/l). The comparative phytochemical and antioxidant characteristics (IC_50_) of the extracts followed the order: CE > AE > EE > ME > HE. Overall, chloroform was the best extraction solvent for *P. ostreatus*. The high content of phenolic compounds, flavonoids, and alkaloids in *P. ostreatus* makes it a rich source of antioxidants and potential candidate for the development of new therapies for a variety of oxidative stress-related disorders.

## Introduction

Natural antioxidants are steadily gaining attention as potent antidote to free radicals generated by rapidly evolving environmental pollutants and unhealthy lifestyles, which disrupt the body's system^1^. The body system has well-designed antioxidant defense mechanisms that prevent oxidative stress^2^. These include enzymatic defenses such as glutathione peroxidase, superoxide dismutase, and catalase^3^ and non-enzymatic defenses, for example, vitamins (A, C, and E, carotenoids, phenols, natural flavonoids, and other antioxidant compounds^4^. However, when the free radicals accumulate, they overwhelmed the body's defensive mechanism and consequently induce oxidative stress^5^.

Recent studies have focused on discovering new exogenous, non-enzymatic, antioxidant sources because of the important role they play in enhancing the body's antioxidant capacity to withstand the influx of reactive oxygen species, thereby preventing oxidative stress^6–8^. Their suggested mechanisms of action, include inhibition of expression or activities of free radical-generating enzymes, increasing the activities or expression of antioxidant enzymes^9^, or directly reacting with ROS to terminate their chain reactions. These effects help to prevent diseases associated with oxidative stress such as inflammation, cancer, aging, cardiovascular diseases, diabetes, and a variety of others^10^.

Edible mushrooms are widely consumed for their therapeutic and nutritional benefits^11^, and have been reported to contain significant levels of minerals in addition to bioactive compounds that exert antioxidant effects^12^. Among the edible mushroom species is *Pleurotus* species. It is an aromatic mushroom, extremely nutritious, widespread, readily available and inexpensive^13–15^. The fungus grows on lingo-cellulosic wastes and could be cultivated without difficulty^16^. Its utilization has been directly linked to improved human health as well as social, cultural, and environmental effects^17^.

The oyster mushroom, *Pleurotus ostreatus,* is an important *Pleurotus species* known for its distinct characteristics, particularly, in terms of its phytochemical contents and antioxidant activities^18^. Different values of phytochemical contents and antioxidant activities have been reported for *P. ostreatu*s. These variations could partly be attributable to the variety of chemicals used to extract the fungus, ranging from polar to nonpolar solvents. This comparative study was designed to evaluate the relative capacities of some common solvents to extract phytochemicals from *P. ostreatus* and the overall nutritional and medicinal value of the fungus for possible product development.

## Materials and methods

All chemicals and reagents used in this study were of analytical grade. The chemicals were obtained from Sigma-Aldrich, Germany and Solarbio Life Science, Beijing, China.

### Sample collection

**Figure 1:**
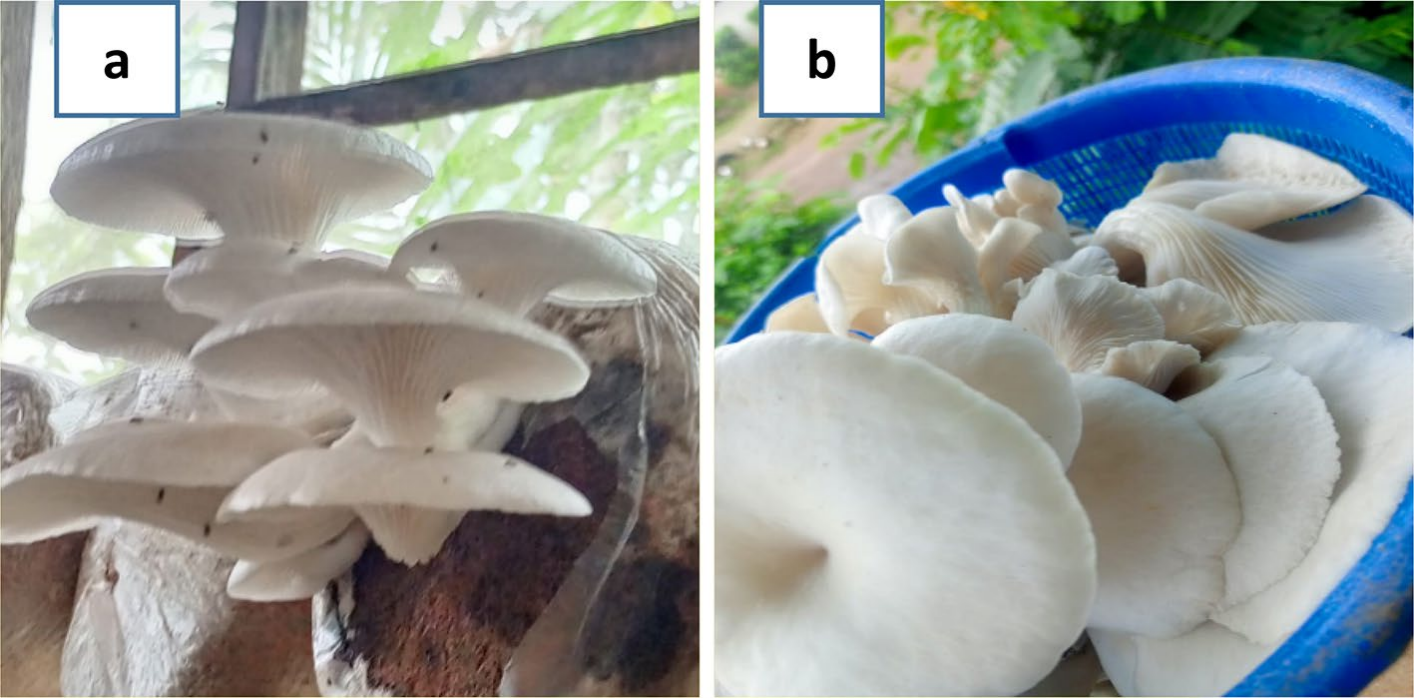
*Pleurotus ostreatus* (oyster mushroom). (**a**) *P. ostreatus* growing on its substrate. (**b**) *P. ostreatus* freshly harvested^15^.

Twenty-five kilogram (25 kg) of fresh *P. ostreatus* (oyster mushroom) were purchased from a local farm in Agbara, Ogun state, Southwest, Nigeria (within the co-ordinates of 6.5114° N, 3.1115° E). The mushroom was identified and authenticated by the Botany department, University of Ibadan, Ibadan, Oyo State, Nigeria (Fig. 1).

### Sample preparation

The *P. ostreatus* sample was thoroughly washed to remove impurities and water residues were removed from its surface with a sterile towel. The sample was then dried in an oven with hot air at 55–65 °C and pulverised with a blender. The resulting powder was weighed at room temperature and stored in airtight containers for subsequent use^19^.

### Sample extraction

The *P. ostreatus* samples were separately extracted with five solvents (distilled water, chloroform, n-hexane, ethanol, and methanol) using the methods described by Zhang et al.^20^ and Mishra et al.^21^. Percentage yield was calculated for each extract. The dried extracts were stored at 4 °C for subsequent tests.

### Qualitative phytochemical analysis

Standard protocols were used to evaluate the various phytochemicals present in the *P. ostreatus* extracts. The secondary metabolites, alkaloids, anthraquinones and betacyanins, anthrocyanins and betacyanins, coumarins, flavonoids, glycosides, tannins, steroids, saponins, terpenoids, phenols, quinones, cardiac glycosides, acids, phlobatannins, and carbohydrates, were assayed using the procedures described by Rahimah et al.^22^, Jiang et al.^23^, Bristy et al.^24^, Hossen et al.^25^, Kaur et al.^26^ and Mishra et al.^21^.

### Quantitative phytochemical analysis

Quantitative phytochemical analysis of saponins, tannins, phenols, flavonoids and alkaloids in the *P. ostreatus* extracts were performed using the methods described by Roghini and Vijayalakshmi^19^ and Mishra et al.^23^.

### In-vitro antioxidant assays

The total antioxidant capacity of *P. ostreatus* extracts was measured by the method described by Adebanke et al.^27^. Free radical scavenging activity (antioxidant capacity) on 2,2-diphenyl-1-picryl-hydrazyl-hydrate (DPPH) was determined using the method described by Mishra et al.^28^ while hydrogen peroxide scavenging capacity was tested by the method described by Salemcity et al.^29^. The' nitric oxide (NO) scavenging activity was determined as described by Awah and Verla^30^ while hydroxyl radical (OH) scavenging activity was measured using the method described by Gulcin et al.^31^ and Adnan et al.^32^. The ferric reducing antioxidant power assay was carried out according to the method reported by Adebanke et al.^27^ while ferrous iron chelating activity was assessed using the method of Vamanu^33^. The ascorbate and guaiacol peroxidase activity of the five *P. ostreatus* extracts were evaluated and compared to quercetin as the assay standard, using the techniques reported by Kumar^34^ and Lepeduš et al.^35^.

### Statistical analysis

Analysis were performed in triplicates and expressed as mean ± SD. Statistical analysis was performed using one-way analysis of variance (ANOVA) to compare the experimental groups and Bonferroni's test was used to find significantly different groups (SPSS for Windows, version 17, SPSS Inc., Chicago, IL). p 0.05 was considered statistically significant.

## Results

### Qualitative phytochemical composition of the five extracts of *P. ostreatus*

The qualitative phytochemical screening of five *P. ostreatus* extracts is shown in Table 1. All the extracts contained carbohydrates, tannins, saponins, flavonoids, alkaloids, anthrocyanins and betacyanins, quinones, cardiac glycosides, phenols, coumarins, and terpenoids while phlobatannins and comarins was found only in the ethanol, methanol, and aqueous extracts. None of the five *P. ostreatus* extracts contained glycosides or acid phytochemicals.

**Table 1:** Qualitative phytochemical composition of *P. ostreatus* extracts.

Extracts	Ethanol	Methanol	Aqueous	Chloroform	n-Hexane
Carbohydrates	+	+	+	+	+
Tannins	+	+	+	+	+
Phlobatannins	+	+	+	−	−
Saponins	+	+	+	+	+
Flavanoids	+	+	+	+	+
Alkaloids	+	+	+	+	+
Anthrocyannins and betacyanins	+	+	+	+	+
Quinones	+	+	+	+	+
Glycosides	−	−	−	−	−
Cardiac glycosides	+	+	+	+	+
Phenols	+	+	+	+	+
Coumarins	+	+	+	−	−
Terpenoids	+	+	+	+	+
Acids	−	−	−	−	−

Present (+) and absent (−).

### Quantitative phytochemical composition of the five extracts of *P. ostreatus*

**Figure 2:**
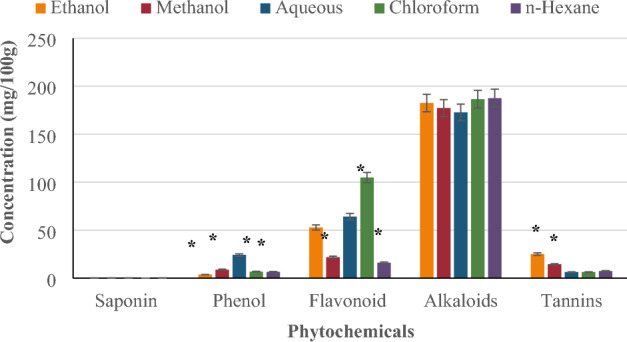
Quantitative phytochemical composition of five extracts of *P. ostreatus.*

Figure 2 shows a plot of the concentrations of saponins, phenols, flavonoids, alkaloids, and tannins in the *P. ostreatus* extracts. The saponin content did not differ significantly (*p < 0.05) across extracts. The n-hexane and methanol extracts contained highest amounts of saponin (0.16 ± 0.00 mg/100 g), while chloroform extract had the least (0.10 ± 0.00 mg/100 g). Both ethanol and methanol extracts contained 0.13 ± 0.00 mg/100 g of saponin. The aqueous extract had significantly higher phenol concentration (*p < 0.05) than the other extracts. The aqueous extract contained the highest amount of phenol (24.14 ± 0.02 mg/100 g), followed by the methanol extract (8.87 ± 0.06 mg/100 g), chloroform extract (6.75 ± 0.08 mg/100 g), n-hexane extract (6.61 ± 0.11 mg/100 g), and ethanol extract (3.71 ± 0.02 mg/100 g). The chloroform extract contained significantly more flavonoids (*p < 0.05) than the other extracts; its flavonoids content of 104.83 ± 29.46 mg/100 g was the highest. This was followed by the aqueous extract (64.17 ± 0.24 mg/100 g), ethanol extract (52.83 ± 0.24 mg/100 g), methanol extract (21.83 ± 0.26 mg/100 g), and n-hexane extract (16.00 ± 4.24 mg/100 g). All the extracts had substantial quantities of alkaloids, with no significant difference (*p < 0.05). The alkaloid content was highest in n-hexane (187.60 ± 0.28 mg/100 g), followed by chloroform (186.50 ± 0.14 mg/100 g), ethanol (182.50 ± 0.14 mg/100 g), methanol (177.20 ± 0.28 mg/100 g), and aqueous extract (172.70 ± 0.14 mg/100 g). Tannin content was highest in the ethanol extract (25.12 ± 0.06 mg/100 g), followed by the methanol extract (14.47 ± 0.01 mg/100 g), n-hexane extract (7.38 ± 0.01 mg/100 g), and lowest in the aqueous (6.31 ± 0.02 mg/100 g) and chloroform extracts (6.31 ± 0.03 mg/100 g). The tannin concentration of ethanol and methanol was significantly greater (*p < 0.05) the content in the other three extracts.

### Antioxidant activities of *P. ostreatus* extracts

#### 1,1-Diphenyl-2-picryl-drazyl (DPPH) radical scavenging activity

**Figure 3:**
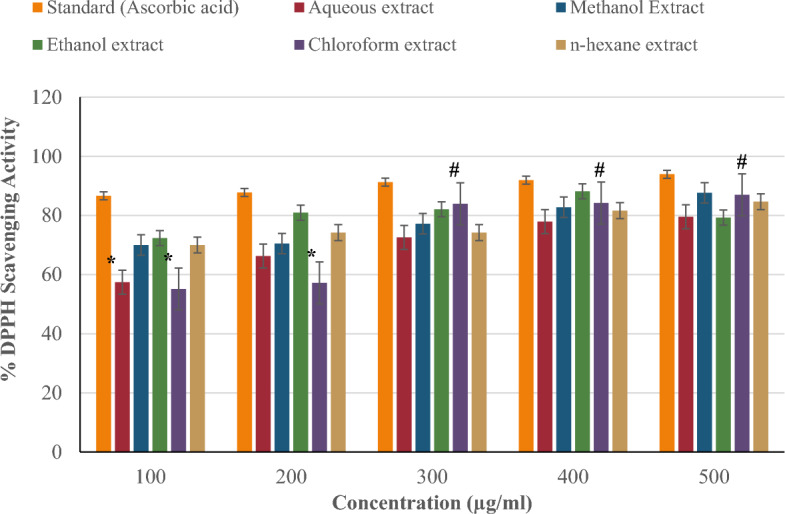
Percentage DPPH scavenging activity of *P. ostreatus* extracts.

Figure 3 compares the DPPH radical scavenging activity of *P. ostreatus* extracts (aqueous, methanol, ethanol, chloroform, and n-hexane) to the standard (ascorbic acid) at concentrations of 100–500 µg/ml. The DPPH radical scavenging activity increased with concentration. Methanol had the highest DPPH radical scavenging activity (87.67%) at 500 µg/ml compared to other extracts. This was followed by chloroform (86.99%), n-hexane (84.65%), aqueous (79.53%), and ethanol (79.30%). However, across all concentrations, none of the extracts demonstrated as much DPPH scavenging activity as ascorbic acid (93.92%).

#### Total antioxidant capacity (TAC)

**Figure 4:**
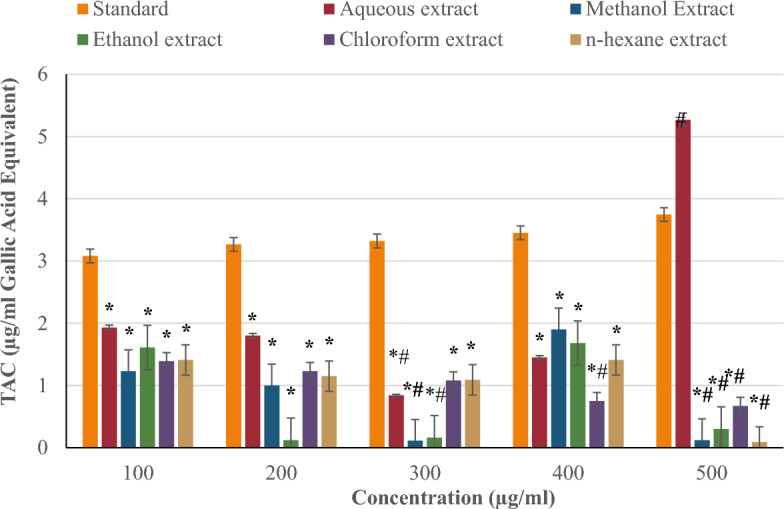
Total antioxidant capacity (TAC) of *P. ostreatus* extracts.

Figure 4 compares the total antioxidant capacity of *P. ostreatus* extracts (aqueous, methanol, ethanol, chloroform, and n-hexane) to the standard (rutin) at concentrations of 100–500 µg/ml. The TAC of the standard (rutin) increased in a concentration-dependent manner, whereas the TAC of the extracts was concentration independent. Among the extracts, aqueous had the highest TAC of 5.27 µg/ml GAE at 500 µg/ml. The methanol extract of 1.90 µg/ml GAE at 400 µg/ml, the ethanol extract of 1.68 µg/ml GAE at 400 µg/ml, the n-hexane extract of 1.41 µg/ml GAE at 100 and 400 µg/ml, and the chloroform extract of 1.39 µg/ml GAE at 100 µg/ml had the lowest TAC value. Across all concentrations, only the aqueous extract had a higher TAC of 5.27 µg/ml GAE when compared to the rutin standard of 3.75 µg/ml GAE at 500 µg/ml.

#### Hydroxyl radical scavenging activity

**Figure 5:**
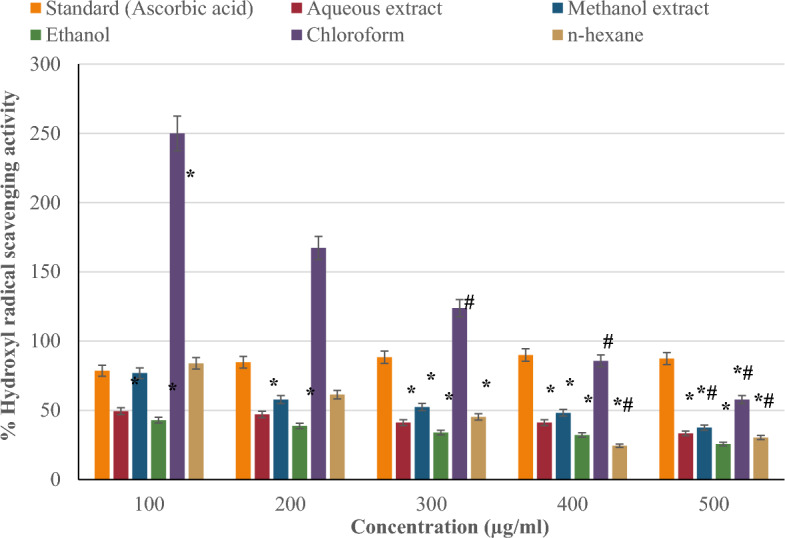
Hydroxyl (OH−) radical scavenging activity of *P. ostreatus* extracts.

Figure 5 compares the hydroxyl radical scavenging capacity of *P. ostreatus* extracts (aqueous, methanol, ethanol, chloroform, and n-hexane) to the standard (ascorbic acid) at concentrations of 100–500 µg/ml. The hydroxyl radical scavenging activity of the standard (ascorbic acid) increased in a concentration-dependent manner except at 500 µg/ml, while the extracts declined with increasing concentration. Chloroform showed the highest hydroxyl radical scavenging activity (250% at 100 µg/ml) compared to other extracts. This was followed by the n-hexane extract with 83.93%, the methanol extract with 76.79%, the aqueous extract with 49.40%, and the ethanol extract with only 42.86%.

#### *Ferric (Fe*^*3*+^*) cyanide reducing potential*

**Figure 6:**
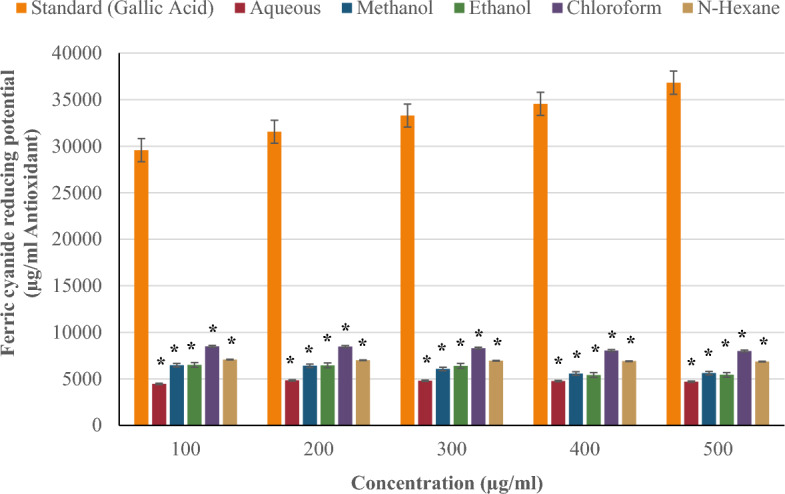
Ferric (Fe^3+^) cyanide reducing potential of *P. ostreatus* extracts.

Figure 6 shows the ferric (Fe^3+^) cyanide reducing potential of the aqueous, methanol, ethanol, chloroform and n-hexane extracts of *P. ostreatus* in comparison with the standard (gallic acid) of the same concentration of 100–500 µg/ml. The ferric cyanide reducing potential of the standard (ascorbic acid) increased in a concentration-dependent manner, but that of the extracts declined with increasing concentration. Chloroform showed the highest ferric cyanide reduction potential (8495 µg/ml) and antioxidant activity (100 µg/ml) among the extracts tested. This was followed by n-hexane (7065 µg/ml), ethanol (6490 µg/ml), and methanol (6470 µg/ml). The aqueous extract has the lowest antioxidant levels. Across all concentrations, none of the extracts showed as much ferric cyanide reduction potential as gallic acid with 36,829 µg/ml antioxidant at 500 µg/ml. There was a significant difference (*p < 0.05) between the ferric cyanide reducing potential of the extracts and the standard at all concentrations.

#### *Ferrous iron (Fe*^*2*+^*) chelating ability*

**Figure 7:**
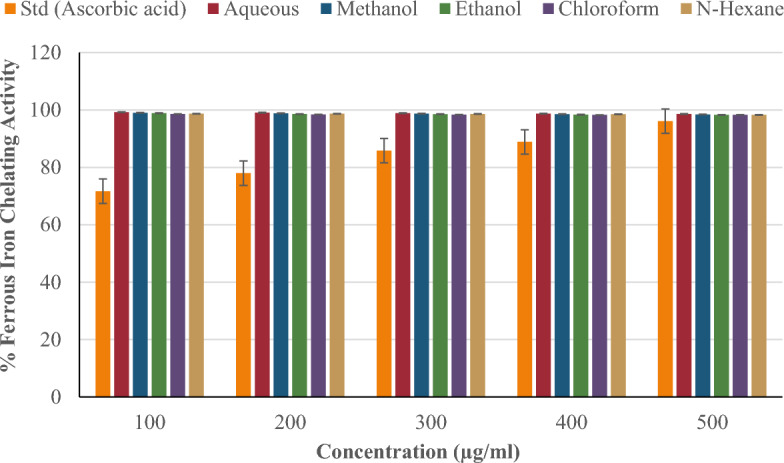
Ferrous Iron (Fe^2+^) Chelating ability of *P. ostreatus* extracts.

Figure 7 compares the ferrous iron (Fe^2+^) chelating activity of *P. ostreatus* extracts (aqueous, methanol, ethanol, chloroform, and n-hexane) to the standard (ascorbic acid) at concentrations of 100–500 µg/ml. The ferrous iron chelating activity of the standard (ascorbic acid) increased in a concentration-dependent manner, whereas the extracts decreased with increasing concentration. Compared to other extracts, aqueous had the highest ferrous iron chelating activity (99.23% at 100 µg/ml). This was followed by the methanol extract (99.02%), the ethanol extract (98.87%), the n-hexane extract (98.69%), and the chloroform extract (98.59%). Across all concentrations, the extracts were more effective at chelating ferrous iron than ascorbic acid. The extracts showed no significant difference (*p < 0.05) in ferrous iron chelating activity compared to the standard at all concentrations.

#### Hydrogen peroxide scavenging activity

**Figure 8:**
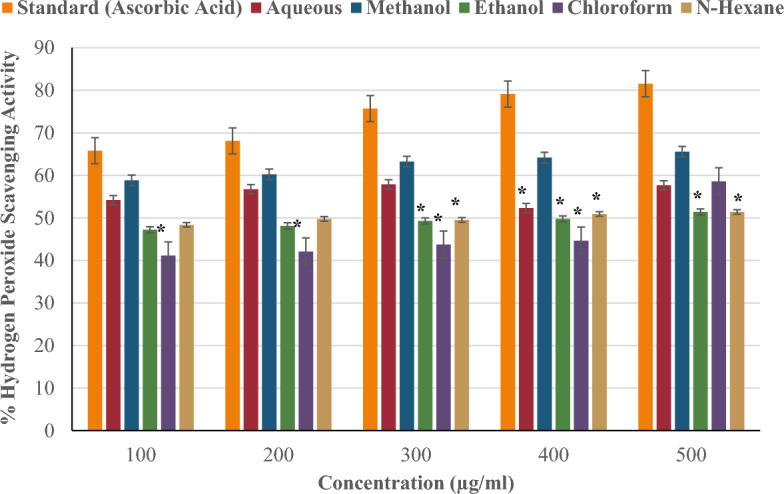
Hydrogen peroxide scavenging activity of *P. ostreatus* extracts.

Figure 8 compares the hydrogen peroxide scavenging activity of *P. ostreatus* extracts (aqueous, methanol, ethanol, chloroform, and n-hexane) to the standard (ascorbic acid) at concentrations of 100–500 µg/ml. The standard (ascorbic acid) and extracts' hydrogen peroxide scavenging activity rose in proportion to their concentrations. Methanol showed the highest hydrogen peroxide scavenging activity (65.58% at 500 µg/ml) compared to other extracts. This was followed by the chloroform extract at 58.60%, the aqueous extract at 57.67%, and the ethanol and n-hexane extracts at 51.39%. At all doses, none of the extracts demonstrated stronger hydrogen peroxide scavenging efficacy than ascorbic acid. The extracts' hydrogen peroxide scavenging activity did not differ significantly (^#^p < 0.05) from that at 100 µg/ml.

#### Nitric oxide radical inhibition

**Figure 9:**
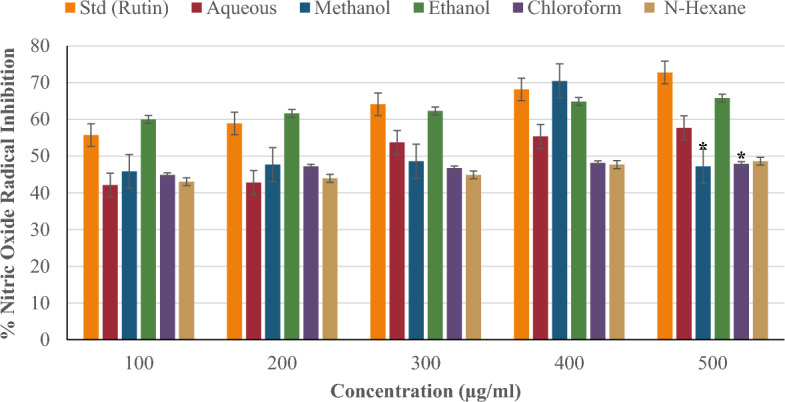
Nitric oxide radical inhibition of *P. ostreatus* extracts.

Figure 9 compares the nitric oxide (NO) radical inhibition of *P. ostreatus* extracts (aqueous, methanol, ethanol, chloroform, and n-hexane) to the standard (rutin) at concentrations ranging from 100 to 500 µg/ml. The nitric oxide radical inhibition of the standard (rutin) and extracts increased in concentration-dependent fashion. At a concentration of 500 µg/ml, ethanol inhibited nitric oxide by 65.81%, surpassing other extracts. This was followed by the aqueous extract (57.67%), the n-hexane extract (48.60%), and the chloroform and methanol extracts (47.91% and 47.21%, respectively). Rutin inhibited nitric oxide radicals more effectively than any other extract at any concentration. The methanol and chloroform extracts inhibited nitric oxide radicals significantly more than the standard at 500 µg/ml (*p < 0.05).

#### Ascorbate peroxidase activity

**Figure 10:**
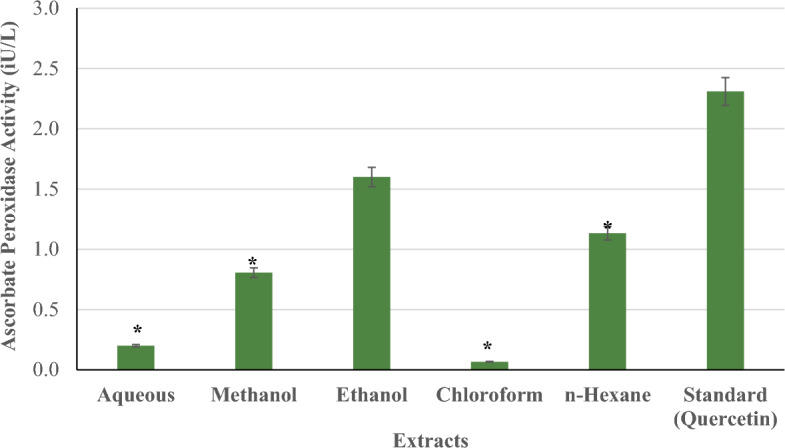
Ascorbate peroxidase activity of *P. ostreatus* extracts.

Figure 10 compares the ascorbate peroxidase activity of *P. ostreatus* extracts (aqueous, methanol, ethanol, chloroform, and n-hexane) to the standard (quercetin) at concentrations of 100–500 µg/ml. Ethanol showed the highest ascorbate peroxidase activity (1.60 iU/l). This was followed by n-hexane extract of 1.13 (iU/l), methanol extract of 0.81 (iU/l), aqueous extract of 0.20 (iU/l), and chloroform extract of 0.06 (iU/l). Across all concentrations, none of the extracts had significantly higher ascorbate peroxidase activity than the standard. Aqueous, methanol, chloroform, and n-hexane extracts had significantly decreased ascorbate peroxidase activity (*p < 0.05) compared to the standard.

#### Guaicol peroxidase activity

**Figure 11:**
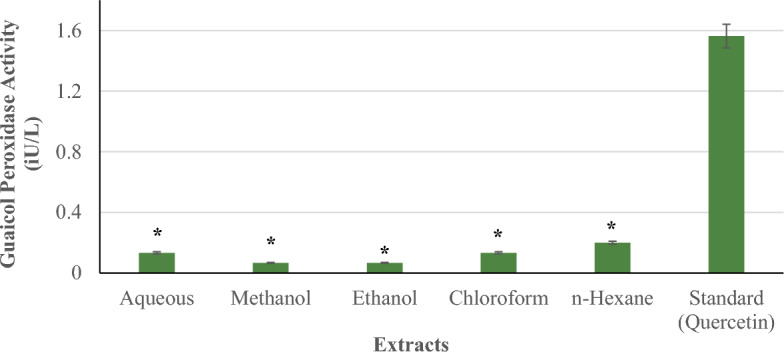
Guaicol Peroxidase activity of *P. ostreatus* extracts.

Figure 11 compares the guaicol peroxidase activity of *P. ostreatus* extracts (aqueous, methanol, ethanol, chloroform, and n-hexane) to the standard (quercetin) at concentrations of 100–500 µg/ml. In comparison to the other extracts, n-hexane showed the greatest guaicol peroxidase activity of 0.20 (iU/l). This was followed by the 0.13 (iU/l) aqueous and chloroform extracts, with the methanol and ethanol extracts coming in last at 0.07 (iU/l). Across all concentrations, none of the extracts had significantly higher guaicol peroxidase activity than the reference. All extracts showed considerably decreased guaicol peroxidase activity (*p < 0.05) compared to the standard.

### Comparative antioxidant activity of the five extracts of *P. ostreatus* based on their IC_50_

Table 2 shows the comparative antioxidant activity of the five *P. ostreatus* extracts based on their IC_50_ values. Ethanol was the most effective solvent for DPPH (IC50: 28.92 µg/ml) and NO (IC_50_: 19.04 µg/ml) scavenging activities. Chloroform was found to be the most effective solvent for decreasing TAC (IC_50_: 27.37 µg/ml) and Fe^3+^ (IC_50_: 11.85 µg/ml). The aqueous solvent had the highest scavenging ability for hydroxyl (IC_50_: 16.52 µg/ml) and H_2_O_2_ (IC_50_: 18.21 µg/ml). n-Hexane extract had the highest Fe^2+^ chelating activity (IC_50_: 10.72 µg/ml).

**Table 2:** IC_50_ of the in-vitro antioxidant activities of five extracts of *P. ostreatus.*

Assays	Extracts (µg/ mL)					
	Standard	Aqueous	Methanol	Ethanol	Chloroform	n-Hexane
DPPH	20.28	54.74	33.75	28.92*	79.35	29.02
TAC	28.72	368.60	595.30	661.6	27.37*^#^	27.63
OH^-^	29.86	16.52*^#^	34.68	20.05	195.20	109.50
Fe^3+^	32.61	57.83	13.07	12.73	11.85*^#^	11.79
Fe^2+^	43.24	11.25	11.26	11.38	11.28	10.72*^#^
H_2_O_2_	34.61	18.21*^#^	22.84	20.13	49.71	18.97
NO*	39.24	52.21	20.68	19.04*^#^	22.07	23.13

Significant values are in bold.

^#^Compares the IC_50_ of the extract to the standard.

*The extract with the least IC_50_.

## Discussion

Reactive oxygen species react with many components in the biological system, creating oxidative damage that requires antioxidants to mitigate^36^. Natural antioxidants empower the body's system to withstand the influx of free radicals and avoid oxidative stress^2,9^. Mushrooms are extremely nutritious foods that contain phenols, flavonoids, and other bioactive components^37^. They have been reported to have anticancer, anti-inflammatory, antidiabetic, and antiaging characteristics, which are mostly attributed to the presence of natural antioxidants^38,39^. The need to highlight the antioxidant benefits of edible mushrooms has grown in significant measure due to the increased importance of antioxidants in combating the rising prevalence of oxidative stress-related illnesses. This study evaluated the phytochemical and antioxidant properties of five *P. ostreatus* extracts to ascertain the optimal extracting solvent for maximizing its medicinal potential.

### Phytochemical composition the five extracts of *P. ostreatus*

The continual imbalance in reactive oxygen species generation and elimination has resulted in several oxidative stress-related diseases and disorders, including cancer, diabetes, aging, cardiovascular diseases, among others^40,41^. Mushrooms synthesize a diverse spectrum of secondary metabolites, including phenols, flavonoids, and other phytochemicals with strong antioxidant effects^38,42,43^. The presence of these phytochemicals in mushrooms indicates that they can help prevent oxidative stress-related diseases and disorders when consumed alone or as food additive^44^. The qualitative phytochemical screening of the five *P. ostreatus* extracts revealed the presence of carbohydrates, tannins, saponins, flavonoids, alkaloids, anthrocyanins and betacyanins, quinones, cardiac glycosides, phenols, coumarins, and terpenoids (Table 1). Mushrooms are exceptionally nutritious foods rich in phenols, flavonoids, and other bioactive compounds^15,21^. These compounds have antioxidant properties and can be taken as an exogenous source of antioxidants^21^. They contribute to the medicinal efficacy of *P. ostreatus*^45,46^. Antioxidants protect cells from free radicals by preventing macromolecule oxidation within the cell^47^. Natural antioxidants serve to prepare the biological system to withstand the influx of free radicals and avoid oxidative stress^48^.

In terms of quantity, alkaloids were the biggest, followed by flavonoids, tannins, and phenols while the lowest was saponins. This supports the findings of Ogidi et al.^49^. Alkaloids are nitrogen-containing chemical compounds found mostly in plants but less so in fungi^50^. The high alkaloid content may be attributed to increased alkaloid production in *P. ostreatus* as a chemical defense, ecological function, or the presence of specific metabolic or biochemical pathways that contribute to the mushroom's overall chemical diversity^50,51^.

Flavonoids are a type of polyphenol with anti-inflammatory and antioxidant properties. They exhibit varying polarity due to the presence of polar hydroxyl and non-polar substituent groups; they donate electrons or hydrogen atoms to free radicals thereby neutralizing them and preventing oxidative stress^52^. The chloroform extract contained the greatest amount of flavonoids when compared to the other *P. ostreatus* extracts. This could be due to the intermediate polarity of chloroform when compared to the other solvents, which allows it to interact more effectively with the cellular structure of *P. ostreatus*, resulting in increased release of lipophilic compounds such as flavonoids. This is consistent with the reports of the superior efficacy of chloroform in extracting flavonoids^53,54^.

Phenols are chemical compounds composed of aromatic hydrocarbon rings and hydroxyl groups. They include polyphenols and flavonoids, which have free radical scavenging and antioxidant properties^55^. The aqueous extract of *P. ostreatus* contained the highest amount of phenols; this may be due to its high polarity and the creation of hydrogen bonds with the hydroxyl groups of the phenols, which increases the solubility and extraction of phenolic compounds. Phenols and flavonoids possess antioxidant properties^56^, with which they quench singlet oxygen and donate hydrogen^57^.

Tannins are toxic when taken in large amounts^48^ because they reduce enzyme activity, interact with proteins, reducing their solubility, palatability, and digestibility^58^. The tannin content of *P. ostreatus* extracts ranged between 6.31 and 25.12 mg/100 g; this is very low when considered with tannin concentrations in plant extracts. Hence, *P. ostreatus* are said to have negligible negative effect on protein and enzyme activity^59^ and are considered safe for human consumption^48^. The ethanol extract of *P. ostreatus* contained the highest amount of tannins when compared to the other extracts. This could be due to ethanol's intermediate polarity that allows for more varied interactions with a wider spectrum of polar and moderately polar molecules, which may boost tannin’s solubility and extraction efficiency via hydrogen bonds.

Saponins froth in aqueous solutions and have a characteristic bitter flavor, which can affect the taste of food when present in large quantities^60^. Saponins cause hemolysis, vomiting, and nausea, and prevents cholesterol absorption, leading to hypercholesterolemia^61^. *P. ostreatus* had a very low saponin content, which could be attributed to its fungal nature, which limits its evolutionary ability to produce saponins when compared to plants. The level of saponin in mushroom species is extremely low, with no detectable effect on the taste of the mushrooms and no ability to destroy sperm cells, rupture red blood cells, or cause nausea and vomiting; however, they can help minimize complications associated with hypocholesterolemia^62^.

### Antioxidant activities of *P. ostreatus* extracts

The antioxidant compounds in *P. ostreatus* were extracted using five different solvents. Their antioxidant properties (in-vitro) were compared to determine the most suitable solvent for extracting the antioxidants. Different methods for assessing antioxidant activity engaged in this study included DPPH scavenging activity, total antioxidant capacity, hydroxyl radical scavenging activity, ferric (Fe^3+^) cyanide chelating activity, ferrous iron (Fe^2+^) chelating ability, hydrogen peroxide scavenging activity, nitric oxide radical inhibition, ascorbate peroxidase activity and guaicol peroxidase activity.

The hydroxyl radical is a free radical with an unpaired electron, making it extremely reactive and capable of destroying important macromolecules like as DNA, protein, and lipids^63^. The extracts' hydroxyl radical scavenging activity was assessed, demonstrating their ability to neutralize hydroxyl radicals by donating electrons or hydrogen atoms. The chloroform extract of *P. ostreatus* exhibited the highest hydroxyl radical scavenging activity. This could be due to its high flavonoid content, which enables the donation of hydrogen atoms from their hydroxyl groups, resulting in hydroxyl radical scavenging. Overall, the hydroxyl radical scavenging activity of the standard (ascorbic acid) rose in a dose-dependent manner whereas the hydroxyl radical scavenging activity of all five *P. ostreatus* extracts declined with increasing concentration. This shows that the extracts are more effective at scavenging hydroxyl radicals at lower concentrations. This could be due to the complex blend of molecules in the extracts with redox cycling, antioxidant, and pro-oxidant properties^64^. These properties may act synergistically at low dosages and antagonistically at higher doses, affecting the overall hydroxyl radical scavenging activity.

DPPH is a stable free radical that contains unpaired electrons. The DPPH scavenging activity assay determined the antioxidants' ability to neutralize DPPH radicals by donating electrons, resulting in a color shift measured with a spectrophotometer^65^. The standard demonstrated greater DPPH scavenging action than any of the *P. ostreatus* extracts, possibly due to its high affinity for DPPH radicals. Similarly, the aqueous study environment gave ascorbic acid an advantage because of its high water solubility compared to the extracts that may be less soluble, thus, limiting their overall effective concentration and scavenging action.

The ferric cyanide reducing potential assay identifies the presence of reducing agents in extracts by donating electrons to convert ferricyanide (Fe^3+^) to ferrocyanide (Fe^2+^), resulting in a color shift that can be detected using a spectrophotometer^66^. The chloroform extract of *P. ostreatus* showed the greatest Fe^3+^ reducing potential, which could be attributable to its high level of flavonoids and phenols that function as reducing agents, rapidly converting Fe^3+^ to Fe^2+^. The ferric cyanide-reducing potential of the standard (gallic acid) increased with concentration, while the extracts dropped in a dose-dependent manner. This could be attributed to the presence of a single well-defined compound in the standard (gallic acid), which could make it more effective than *P. ostreatus* extracts that contain a diverse range of compounds with varying redox capacities, thereby, influencing the concentration–response relationship.

In contrast to the concentration-dependent decrease in the ferric cyanide potential of *P. ostreatus* extracts, the ferrous iron chelating activity increased with concentration. The test examined the extracts' ability to bind or chelate ferrous ions. This is important to keep Fe^2+^ from participating in the Fenton reaction. The Fenton reaction involves the reaction of Fe^2+^ and hydrogen peroxide, resulting in Fe^3+^, hydroxyl radicals, and hydroxide ions. This process produces oxidative stress and a number of degenerative disorders^67^. At all concentrations, the five *P. ostreatus* extracts outperformed the standard (ascorbic acid) for iron chelation. This could be explained by the diverse chelating components in the extracts, including phenols, flavonoids, and other phytochemicals, compared to the standard (ascorbic acid). Similarly, the extract's chelating compounds may have complementary chelating characteristics, such as a stronger affinity for ferrous ions than ascorbic acid.

The hydrogen peroxide (H_2_O_2_) scavenging capacity of the *P. ostreatus* extracts determined by their ability to react with H_2_O_2_ to produce water and oxygen. H_2_O_2_ neutralization prevents the production of hydroxyl radicals in the body, which can harm biomolecules via the Fenton reaction^68^. The ability of the standard and extracts to scavenge H_2_O_2_ improved with increasing concentration. At all doses, ascorbic acid outperformed the extracts in terms of H_2_O_2_ scavenging activity. This could be attributed to the high antioxidant activity, accessibility, and reactivity of ascorbic acid with H_2_O_2_ compared to the extracts, which contain several antioxidant components that may not act synergistically to exert considerable scavenging action.

The nitric oxide synthase enzyme in cells generates nitric oxide, a signaling molecule that also serves as a free radical. During the immune response, these cells, particularly immune cells like macrophages, generate nitric oxide^69^. However, excessive nitric oxide production causes inflammation and oxidative stress by interacting with molecular oxygen, producing nitrite (NO_2_^-^) and other nitrogen oxide species^70^. The ability of the *P. ostreatus* extracts to inhibit nitric oxide radical generation was investigated. The nitric oxide radical inhibition activity of the extracts and standard (rutin) increased in a concentration-dependent manner. At higher concentrations of 300–500 µg/ml, rutin inhibited nitric oxide radicals more than all *P. ostreatus* extracts. It was less efficacious compared to ethanol extract at lower doses (100–200 µg/ml). This shows that, at lower concentrations, the compounds in the ethanol extract may be more effective and preferential for nitric oxide inhibition than the standard (rutin). However, at higher concentrations, a reverse trend may occur due to an overload or saturation effect of the molecules in the ethanol extract, resulting in pro-oxidant activity and declining effects. Rutin, on the other hand, may not be prone to such interference being a single pure chemical.

The total antioxidant capacity (TAC) examined the ability of *P. ostreatus* extracts to quench reactive oxygen species and free radicals, both of which contribute to oxidative stress. The TAC of the standard (rutin) and aqueous extract increased with increasing concentration, whereas that of the *P. ostreatus* extracts decreased in a concentration-dependent manner. This could be due to the high phenol and flavonoid content of the aqueous extracts, which could interact to boost TAC when compared to other extracts.

Ascorbate peroxidase and guaicol peroxidase enzymes are essential components of the cellular antioxidant system, helping to protect cells from oxidative damage by neutralizing hydrogen peroxide^71^. The standard (quercetin) had much higher ascorbate and guaicol peroxidase activity than any *P. ostreatus* extract. In comparison, *P. ostreatus* extracts, with the exception of chloroform, have stronger ascorbate peroxidase activity than guaicol peroxidase activity. This could be due to the presence of chemicals in the extracts that work better as substrates for ascorbate peroxidase than guaicol peroxidase.

### Comparative solvent suitability based on antioxidant and phytochemical profiles

IC_50_ is defined as the amount of an antioxidant-containing material required to scavenge 50% of a radical. An antioxidant substance or extract is said to be more effective at scavenging radicals when it has a lower IC_50_, hence, the lower the IC_50_ value, the higher the antioxidant activity^72^.

Chloroform had the highest hydroxyl radical scavenging and ferric cyanide reduction activity at 250 µg/ml and 8495 µg/ml, respectively. It also gave the second highest DPPH scavenging activity, hydrogen peroxide scavenging activity, and guaicol peroxidase activity of 86.99%, 58.61%, and 0.13 iU/l, respectively. Based on its IC_50_, the primary antioxidant mechanisms are ferric cyanide-reducing power antioxidant and total antioxidant capacity.

The aqueous extract had the highest total antioxidant capacity and ferric ion chelating activity of 5.27 µg/ml GAE and 99.23% respectively. Similarly, it had the second highest nitric oxide inhibition activity, hydrogen peroxide scavenging activity, and guaicol peroxidase activity of 5.67%, 57.91%, and 0.13 iU/l, respectively. The principal antioxidant mechanisms, based on its IC_50_, are hydroxyl radical and hydrogen peroxide scavenging activity.

The methanol extract exhibited the highest DPPH and hydrogen peroxide scavenging activity of 87.67% and 65.58% respectively. It also had the second highest TAC and ferrous iron chelating activity of 1.90 µg/ml GAE and 99.02%, respectively. The ethanol extract inhibited nitric oxide by 65.81% and had ascorbate peroxidase activity of 1.60 iU/l. The ethanol extract's IC_50_ data revealed that its main antioxidant mechanisms are DPPH and nitric oxide scavenging activities.

The n-hexane extract exhibited the greatest guaicol peroxidase of 0.20iU/l, as well as the second highest ferric cyanide reduction activity and hydroxyl radical scavenging activity of 7065 µg/ml antioxidant and 83.93%, respectively. The IC_50_ values of the extract showed that its primary antioxidant mechanism is ferric ion chelation potential.

## Conclusion

The five extraction solvents used in this study yielded variable amounts of diverse phytochemicals present in *P. ostreatus* and also different levels of antioxidant activities. The chloroform, aqueous, n-hexane, and ethanol extracts respectively gave the highest amount of flavonoids, phenolic compounds, alkaloids, and tannins, whereas both methanol and n-hexane extracts had the same and best content of saponins.

The primary antioxidant mechanisms of the *P. ostreatus* extracts included free radical scavenging, Fe^3+^ reduction, and Fe^2+^ chelation. The study demonstrated the ability of *P. ostreatus* to scavenge a wide range of free radicals, including DPPH, hydroxyl radical, nitric oxide, and hydrogen peroxide, suggesting a wide range of medical and physiological uses in view of its capacity to protect important cell macromolecules, reduce free radical generation, and prevent oxidative stress (Supplementary).

The in-vitro antioxidant experiments showed that chloroform was the best extracting solvent for *P. ostreatus* as evidenced by its best antioxidant properties and phytochemical content when compared to the other extracts. In conclusion, *P. ostreatus* has been shown to be a rich source of natural antioxidants and nutraceuticals, with chloroform acting as an excellent extraction solvent.

### Supplementary Information

Supplementary Tables.

## Data Availability

All data and materials used or generated in this study are available and may be provided on request by the corresponding author.
