# Supplementary material for: Comparative antioxidant activity and phytochemical content of five extracts of Pleurotus ostreatus (oyster mushroom)
Source: Sci Rep. 2024 Feb 15;14:3794. doi: 10.1038/s41598-024-54201-x (PMC10869810; doi:10.1038/s41598-024-54201-x)
Supplement: Supplementary file 1 — Supplementary Tables. [file 41598_2024_54201_MOESM1_ESM.docx]

**COMPARATIVE ANTIOXIDANT ACTIVITY AND PHYTOCHEMICAL CONTENT OF FIVE EXTRACTS OF *Pleurotus ostreatus* (OYSTER MUSHROOM)**

*Magdalene Eno Effiong^1,2*^, Israel Sunmola Afolabi^1,3^, Umeokwochi, Chidinma Precious ^1^ and Shalom Nwodo Chinedu^1,3^*

*Corresponding author: [magdalene.effiongpgs@stu.cu.edu.ng](mailto:magdalene.effiongpgs@stu.cu.edu.ng)

**Supplementary Tables**

Table 1: Quantitative phytochemical composition of five extracts of *Pleurotus ostreatus*

| Extracts (mg/100g) | Ethanol | Methanol | Aqueous | Chloroform | n-Hexane |
| --- | --- | --- | --- | --- | --- |
| Saponin | 0.13 ± 0.00 | 0.16 ± 0.00* | 0.13 ± 0.00 | 0.10 ± 0.00 | 0.16 ± 0.00* |
| Phenol | 3.71 ± 0.02 | 8.87 ± 0.06 | 24.14 ± 0.02* | 6.75 ± 0.08 | 6.61 ± 0.11 |
| Flavonoid | 52.83 ± 0.24 | 21.83 ± 0.26 | 64.17 ± 0.24 | 104.83 ± 29.46***** | 16.00 ± 4.24 |
| Alkaloids | 182.50 ± 0.14 | 177.20 ± 0.28 | 172.70 ± 0.14 | 186.50 ± 0.14 | 187.60 ± 0.28* |
| Tannins | 25.12 ± 0.06* | 14.47 ± 0.01 | 6.31 ± 0.02 | 6.31 ± 0.03 | 7.38 ± 0.01 |

**Table 2: Percentage DPPH scavenging activity of *Pleurotus ostreatus* extracts**

| Concentration (µg/mL) | Standard (Ascorbic acid) | Aqueous extract | Methanol Extract | Ethanol extract | Chloroform extract | n-hexane extract |
| --- | --- | --- | --- | --- | --- | --- |
| 100 | 86.63 ± 0.23 | 57.44 ± 0.33***** | 70.00 ± 0.33 | 72.33 ± 0.99 | 55.12 ± 1.64***** | 70.00 ± 0.33 |
| 200 | 87.75 ± 0.23 | 66.28 ± 0.33 | 70.47 ± 0.33 | 80.93 ± 0.66 | 57.21 ± 1.32***** | 74.19 ± 2.30 |
| 300 | 91.27 ± 0.45 | 72.59 ± 0.66 | 77.21 ± 5.92 | 82.09 ± 0.33 | 83.95 ± 0.33**^#^** | 74.19 ± 4.28 |
| 400 | 91.91 ± 0.45 | 77.91 ± 0.99 | 82.79 ± 0.66 | 88.14 ± 0.33 | 84.19 ± 2.63**^#^** | 81.63 ± 0.33 |
| 500 | 93.92 ± 0.57 | 79.53 ± 0.66 | 87.67 ± 0.33 | 79.30 ± 0.33 | 86.99 ± 1.31**^#^** | 84.65 ± 0.66 |

**Table 3: Total Antioxidant Capacity of *Pleurotus ostreatus* extracts**

| Concentration (µg/mL) | Standard (Rutin) | Aqueous extract | Methanol Extract | Ethanol extract | Chloroform extract | n-hexane extract |
| --- | --- | --- | --- | --- | --- | --- |
| 100 | 3.08 ± 0.00 | 1.93 ± 0.14* | 1.23 ± 0.05* | 1.61 ± 0.02* | 1.39 ± 0.01* | 1.41 ± 0.01* |
| 200 | 3.27 ± 0.01 | 1.80 ± 0.01* | 1.00 ± 0.13* | 0.12 ± 0.05***^#^** | 1.23 ± 0.15* | 1.15 ± 0.03* |
| 300 | 3.32 ± 0.57 | 0.84 ± 0.01***^#^** | 0.11 ± 0.09***^#^** | 0.16 ± 0.11***^#^** | 1.08 ± 0.05* | 1.09 ± 0.06* |
| 400 | 3.45 ± 0.01 | 1.45 ± 0.02* | 1.90 ± 0.01* | 1.68 ± 0.02* | 0.75 ± 0.05***^#^** | 1.41 ± 0.01* |
| 500 | 3.75 ± 0.00 | 5.27 ± 0.31**^#^** | 0.12 ± 0.01***^#^** | 0.30 ± 0.02***^#^** | 0.67 ± 0.03***^#^** | 0.09 ± 0.03***^#^** |

Table 4: Hydroxyl (OH-) radical scavenging activity of *Pleurotus ostreatus* extracts

| Concentration (µg/mL) | Standard (Ascorbic acid) | Aqueous extract | Methanol extract | Ethanol | Chloroform | n-hexane |
| --- | --- | --- | --- | --- | --- | --- |
| 100 | 78.57 ± 0.92 | 49.40 ± 0.84* | 76.79 ± 0.84 | 42.86 ± 1.68* | 250.00 ± 1.68* | 83.93 ± 0.84 |
| 200 | 84.74 ± 1.38 | 47.02 ± 0.84* | 57.74 ± 7.58 | 38.69 ± 0.84* | 167.26 ± 0.84* | 61.31 ± 0.84 |
| 300 | 88.31 ± 0.92 | 41.07 ± 0.84* | 52.38 ± 1.68* | 33.93 ± 0.84* | 123.81 ± 1.68**^#^** | 45.24 ± 1.68* |
| 400 | 89.94 ± 0.46 | 41.07 ± 0.84* | 48.21 ± 4.21* | 32.14 ± 1.68* | 85.71 ± 3.37**^#^** | 24.41 ± 7.58***^#^** |
| 500 | 87.34 ± 1.38 | 33.33 ± 1.68* | 37.50 ± 0.84***^#^** | 25.60 ± 4.21* | 57.74 ± 0.84***^#^** | 30.36 ± 0.84***^#^** |

**Table 5: Ferric (Fe^3+^) cyanide reducing potential** **of *Pleurotus ostreatus* extracts**

| Concentration (µg/mL) | Standard  (Gallic acid) | Aqueous extract | Methanol extract | Ethanol | Chloroform | n-hexane |
| --- | --- | --- | --- | --- | --- | --- |
| 100 | 29575 ± 1272.10 | 4455 ± 7.07* | 6470 ± 14.14* | 6490 ± 28.28* | 8495 ± 35.36* | 7065 ± 35.36* |
| 200 | 31552 ± 509.12 | 4830 ± 28.28* | 6405 ± 7.07* | 6455 ± 7.07* | 8465 ± 7.07* | 7005 ± 35.36* |
| 300 | 33291 ± 84.85 | 4800 ± 14.14* | 6065 ± 7.07* | 6400 ± 14.14* | 8285 ± 7.07* | 6950 ± 14.14* |
| 400 | 34551 ± 338.70 | 4765 ± 7.07* | 5565 ± 7.07* | 5415 ± 7.07* | 8035 ± 7.07* | 6900 ± 42.43* |
| 500 | 36829 ± 1187.20 | 4695 ± 21.21* | 5615 ± 7.07* | 5430 ± 56.57* | 7990 ± 14.14* | 6855 ± 7.07* |

**Table 6: Ferrous Iron (Fe^2+^) % Chelating ability of *Pleurotus ostreatus* extracts**

| Concentration (µg/mL) | Standard | Aqueous extract | Methanol extract | Ethanol | Chloroform | n-hexane |
| --- | --- | --- | --- | --- | --- | --- |
| 100 | 71.70 ± 2.23 | 99.23 ± 0.06 | 99.02 ± 0.06 | 98.87 ± 0.15 | 98.59 ± 0.06 | 98.69 ± 0.03 |
| 200 | 77.97 ± 1.28 | 99.06 ± 0.18 | 98.84 ± 0.12 | 98.57 ± 0.09 | 98.40 ± 0.03 | 98.69 ± 0.03 |
| 300 | 85.81 ± 2.00 | 98.87 ± 0.03 | 98.72 ± 0.06 | 98.55 ± 0.12 | 98.35 ± 0.03 | 98.61 ± 0.03 |
| 400 | 88.86 ± 0.73 | 98.72 ± 0.06 | 98.55 ± 0.06 | 98.31 ± 0.03 | 98.27 ± 0.03 | 98.50 ± 0.06 |
| 500 | 96.09 ± 0.32 | 98.57 ± 0.03 | 98.44 ± 0.03 | 98.33 ± 0.12 | 98.33 ± 0.12 | 98.27 ± 0.09 |

**Table 7: Hydrogen peroxide scavenging activity of *Pleurotus ostreatus* extracts**

| Conc. (µg/mL) | Standard  (Ascorbic acid) | Aqueous extract | Methanol extract | Ethanol | Chloroform | n-hexane |
| --- | --- | --- | --- | --- | --- | --- |
| 100 | 65.79 ± 0.47 | 54.19 ± 0.33 | 58.84 ± 0.33 | 47.21 ± 0.33 | 41.16 ± 0.99* | 48.37 ± 0.66 |
| 200 | 68.10 ± 1.27 | 56.74 ± 0.66 | 60.23 ± 0.33 | 48.14 ± 0.99 | 42.09 ± 0.99* | 49.77 ± 1.32 |
| 300 | 75.70 ± 2.25 | 57.91 ± 0.33 | 63.26 ± 0.66 | 49.30 ± 0.66* | 43.72 ± 0.66* | 49.54 ± 0.99* |
| 400 | 79.11 ± 1.43 | 52.33 ± 0.33* | 64.19 ± 0.66 | 49.78 ± 1.32* | 44.65 ± 0.66* | 50.93 ± 0.33* |
| 500 | 81.56 ± 0.77 | 57.67 ± 0.66 | 65.58 ± 1.32 | 51.40 ± 0.99* | 58.61 ± 0.66 | 51.40 ± 0.33* |

**Table 8: Nitric oxide radical inhibition of *Pleurotus ostreatus* extracts**

| Conc.  (µg/mL) | Standard | Aqueous extract | Methanol extract | Ethanol | Chloroform | n-hexane |
| --- | --- | --- | --- | --- | --- | --- |
| 100 | 55.72 ± 0.55 | 42.09 ± 0.33 | 45.81 ± 0.33 | 60.00 ± 0.00 | 44.88 ± 0.33 | 43.02 ± 0.33 |
| 200 | 58.92 ± 0.68 | 42.79 ± 0.66 | 47.67 ± 0.33 | 61.63 ± 0.33 | 47.21 ± 0.33 | 43.95 ± 0.99 |
| 300 | 64.11 ± 0.31 | 53.72 ± 0.33 | 48.61 ± 0.33 | 62.33 ± 0.66 | 46.74 ± 0.33 | 44.88 ± 0.33 |
| 400 | 68.17 ± 1.50 | 55.35 ± 1.32 | 48.97 ± 4.21 | 54.88 ± 0.99 | 48.14 ± 0.99 | 47.67 ± 0.33 |
| 500 | 72.78 ± 0.47 | 57.67 ± 1.32 | 47.21 ± 0.33* | 65.81 ± 0.33 | 47.91 ± 0.66* | 48.61 ± 0.33 |

**Table 9: Ascorbate peroxidase activity of *Pleurotus ostreatus* extracts**

| Extracts | Ascorbate Peroxidase (iU/L) |
| --- | --- |
| Aqueous | 0.20 ± 0.000* |
| Methanol | 0.81 ± 0.001* |
| Ethanol | 1.60 ± 0.000 |
| Chloroform | 0.06 ± 0.007* |
| n-Hexane | 1.13 ± 0.003* |
| Standard (Quercetin) | 2.31 ± 0.001 |

**Table 10: Guaicol Peroxidase activity of *Pleurotus ostreatus* extracts**

| Extracts | Guaicol Peroxidase (iU/L) |
| --- | --- |
| Aqueous | 0.13 ± 0.003* |
| Methanol | 0.07 ± 0.001* |
| Ethanol | 0.07 ± 0.002* |
| Chloroform | 0.13 ± 0.002* |
| N Hexane | 0.20 ± 0.000* |
| Standard (Quercetin) | 1.56 ± 0.003` |
